# Supplementary material for: Genotypic Diversity, Antibiotic Resistance, and Virulence Phenotypes of Stenotrophomonas maltophilia Clinical Isolates from a Thai University Hospital Setting
Source: Antibiotics (Basel). 2023 Feb 18;12(2):410. doi: 10.3390/antibiotics12020410 (PMC9951947; doi:10.3390/antibiotics12020410)
Supplement: Supplementary file 1 [file antibiotics-12-00410-s001.zip › antibiotics-2118717-supplementary.pdf]

Supplementary Materials

Supplementary Tables

Table S1. MDR and non-MDR phenotypes of *S. maltophilia* isolates.

Antibiotic resistance (No. of isolates)	MDR phenotype * No. of isolates (%)	Correlation[a]	Significance[b]	Non-MDR phenotype No. of isolates (%)	Correlation	Significance
TMP/SMX resistance (15)	13 (86.67)	*r* = 0.749	P < 0.001	2 (13.33)	*r* = -0.616	P < 0.001
LEV resistance (18)	13 (72.22)	*r* = 0.504	P < 0.001	5 (27.78)	*r* = -0.524	P < 0.001
CAZ resistance (157)	20 (12.74)	*r* = 0.108	P = 0.128	137 (87.26)	*r* = -0.098	P = 0.167
C resistance (31)	20 (64.52)	*r* = 0.696	P < 0.001	11 (35.48)	*r* = -0.625	P < 0.001
Non-resistance (37)	0	-	-	37 (100)	r = -0.910	P < 0.001
MDR (20)	20 (100)	r = -0.910	P < 0.001	0	-	-

MDR phenotype was considered by the isolate that was resistance to ≥ three antibiotics; [a] Correlation matrix exhibited the correlation between MDR/non- MDR phenotypes and antibiotic resistance groups; The relationships were determined by Spearman correlation coefficients; Positive correlation was shown in high r value and negative correlation was shown in minus r value; [b]A statistically significance of the correlation was set at $p < 0.05$. TMP/SMX, trimethoprim/sulfamethoxazole; LEV, levofloxacin; CAZ, ceftazidime; C, chloramphenicol; MDR, multidrug resistance.

Table S2. Primer pairs used in detection of antibiotic resistance genes among *S. maltophilia* isolates

Primer name	Primer nucleotide sequence (5' → 3')	Annealing temp. (°C)	Reference
smeF forward	CCAACGCGGATCGTGATATC	55	[25]
smeF reverse	TGCTCATCCAGGCTGACATTC		
bla_{L1} forward	CGGCATGCCACAGATGG	59	[64]
bla_{L1} reverse	GCAGCACCGCCGTTTCT		
bla_{L2} forward	CGACAATGCCGCAGCTAACC	59	[65]
bla_{L2} reverse	CAGAAGCAATTAATAACGCCC		
intI1 forward	TTCGCAACTACACCATGAAC	48	[50]
intI1 reverse	CAGCGCGACTCCTTGTACTT		
sul1 forward	ATGGTGACGGTGTTCGGCATT CTGA	64	[30]
sul1 reverse	CTAGGCATGATCTAACCCTCG GTCT		
*sul*2 forward	GAATAAATCGCTCATCATTTTCGG	64	[66]
sul2 reverse	CGAATTCTTGCGGTTTCTTTCAGC		
floR forward	TCGACATCCTGGCTTCACTG	59	[67]
floR reverse	ATTACAAGCGCGACAGTGGGC		
mfsA forward	TCTGACCCGTATGACCCCGA	59	[68]
mfsA reverse	GCGGCGCTACACCCGTCG		
